# Less Expansion of Short-Pulse Laser Scars in Panretinal Photocoagulation for Diabetic Retinopathy

**DOI:** 10.1155/2018/9371895

**Published:** 2018-04-19

**Authors:** Masahiko Higaki, Miho Nozaki, Munenori Yoshida, Yuichiro Ogura

**Affiliations:** Department of Ophthalmology and Visual Science, Nagoya City University Graduate School of Medical Sciences, Nagoya 467-8601, Japan

## Abstract

**Purpose:**

To compare the expansion rates of laser photocoagulation scars between the conventional laser and short-pulse laser using fundus autofluorescence (FAF).

**Methods:**

Retrospective chart review. Conventional laser was performed on 6 eyes of 6 patients, and short-pulse laser was performed on 11 eyes of 8 patients with diabetic retinopathy. FAF images were obtained by Optos® 200Tx (Optos, Dunfermline, Scotland, UK) at 1, 3, 6, and 12 months after treatment. The average area of 20 photocoagulation scars was measured by using ImageJ software. The expansion rates were calculated from the proportion of the averaged area against the optic disc area. Regression of retinopathy and central macular thickness were also evaluated.

**Results:**

The expansion rates of the conventional laser scars compared with the size at 1 month after treatment were 1.12 ± 0.08 (3 M), 1.27 ± 0.12 (6 M), and 1.39 ± 0.11 (12 M). The expansion rates of the short-pulse laser scars were 1.04 ± 0.05 (3 M), 1.09 ± 0.04 (6 M), and 1.13 ± 0.05 (12 M). The expansion rates of the short-pulse laser were significantly lower than those of the conventional laser (*p* < 0.01).

**Conclusion:**

FAF images were useful to evaluate the changes in the photocoagulation scar sizes. The scars with the short-pulse laser showed lower expansion rates than those of the conventional laser.

## 1. Introduction

Diabetic retinopathy is the leading cause of blindness in the working population of the Western world [1]. Although panretinal photocoagulation (PRP) is the standard therapy for reducing the activity of diabetic retinopathy [2], PRP sometimes results in decreased visual acuity due to PRP-induced macular edema [3–5]. Recently, short-pulse pattern scan laser system (PASCAL® Streamline, Topcon Medical Laser systems, Santa Clara, CA, USA) has been developed [6, 7], and it is known that short-pulse laser treatment is quicker, generates less heat, and is less painful to eyes than the conventional laser treatment [6]. Moreover, some reports indicate that short-pulse laser treatment induces less inflammation, fewer inflammatory cytokines in the sensory retina, and less macular thickening in patients with diabetic retinopathy than the conventional pulse duration [2–4, 8, 9].

Despite these advantages of the short-pulse laser, some studies indicate that short-pulse laser is less effective than the conventional laser treatment in treatment for the high-risk proliferative diabetic retinopathy. They suggested that the reason for the differences was that the total area of PRP scars generated by the conventional laser exceeded that of short-pulse laser although both groups were treated with the same number of laser spots [5, 10]. The photocoagulation scars performed by the conventional laser have a tendency to expand after treatment [5, 8, 11–14]. However, some reports revealed that the expansion rate of photocoagulation scars performed by the short-pulse laser is lower than that of the conventional laser [8, 12, 15]. In these reports, the laser scars were evaluated by using examination including color fundus photographs, fluorescein angiograms, and infrared images [8, 12] or OCT [16].

FAF imaging is a noninvasive technique used to assess retinal pigment epithelial (RPE) cells and now widely used to evaluate age-related macular degeneration [17], retinitis pigmentosa [18], and other chorioretinal diseases. FAF signals increase with lipofuscin accumulation in RPE cells and decrease with RPE atrophy [19]. Analysis of FAF is an effective method to observe the functions of the RPE cells. Since retinal laser photocoagulation targets to RPE, FAF analysis after laser photocoagulation is thought to be an effective method to evaluate the RPE alterations and efficacy of laser photocoagulation. Although Muqit et al. already evaluated laser photocoagulation scars using FAF, they compared the FAF changes between the conventional laser and short-pulse laser only for 4 weeks [15], or they only followed FAF changes of 2 cases treated with short-pulse laser PRP [16].

In this study, we aimed to compare the FAF changes between the conventional laser and short-pulse laser in treatment of diabetic retinopathy, in terms of laser scar expansion rates and disease regression for 12 months.

## 2. Methods

This study was a retrospective cohort study. This study was approved by the Institutional Review Board of Nagoya City University Graduate School of Medical Science, conducted in accordance with the ethical standards stated in the 1964 Declaration of Helsinki.

All patients were treated at Nagoya City University Hospital between September 2013 and February 2015. All patients were followed for at least 12 months after laser photocoagulation. The patients with media opacities such as corneal opacity, cataract, and vitreous hemorrhage, which may influence the FAF images, were excluded.

We evaluated the best corrected visual acuity (BCVA), the central macular thickness (CMT) in OCT (Cirrus HD-OCT 4000, Carl Zeiss Meditec, Dublin, CA, Germany), the regressions of neovascularization, and the expansion of photocoagulation scars in 17 eyes of 12 patients with diabetic retinopathy (PDR; 5 eyes, NPDR; 12 eyes).

The BCVA was measured with a Japanese standard decimal visual acuity chart, and decimal BCVA was calculated using the logarithm of the minimum angle of resolution (logMAR) scale.

**Figure 1:**
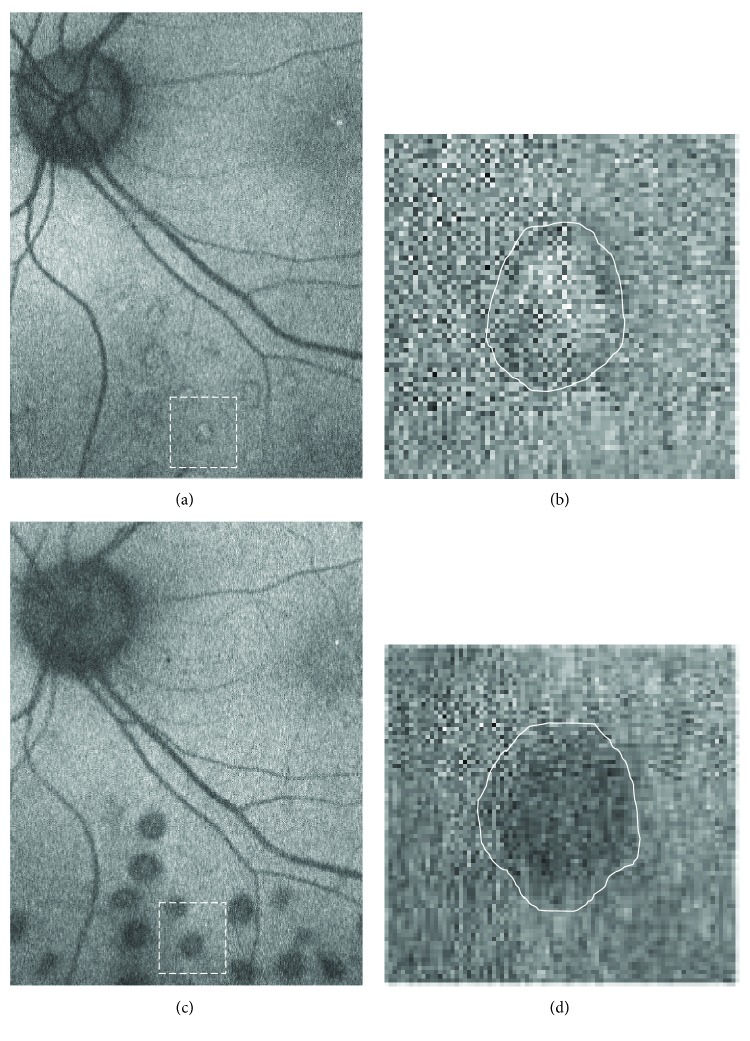
Representative images of fundus autofluorescence (FAF) in the conventional laser group. The images were taken 1 month after laser treatment (a, b) and 12 months after treatment (c, d). Twenty laser scars near the vascular arcade were measured using the digital image analysis software ImageJ on each visit. Higher magnification of the area surrounded by white-dashed line was shown in (b) and (d). White line indicated the outline of FAF laser scars for measurement (b, d). High magnification images show the changes of laser scars from hyperautofluorescent at 1 months (b) to hypoautofluorescent at 12 months after laser treatment (d).

**Figure 2:**
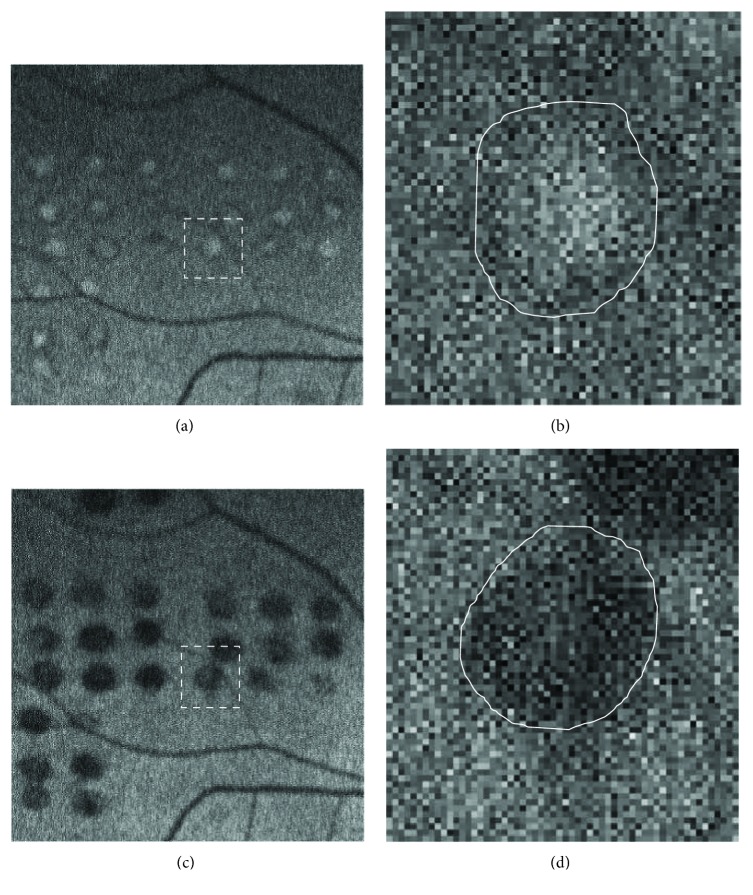
Representative images of FAF in the short-pulse laser group. The images were taken 1 month after laser treatment (a, b) and 12 months after treatment (c, d). Twenty laser scars near the vascular arcade were measured using the digital image analysis software ImageJ on each visit. Higher magnification of the area surrounded by white-dashed line was shown in (b) and (d). White line indicated the outline of FAF laser scars for measurement (b, d). High magnification images show the changes of laser scars from hyperautofluorescent at 1 month (b) to hypoautofluorescent at 12 months after laser treatment (d).

FAF images were taken by Optos 200Tx at 1, 3, 6, and 12 months after treatment. We measured the pixel sizes of an optic disc and 20 laser scars near the vascular arcade on each visit using the digital image analysis software ImageJ (developed by Wayne Rasband, National Institutes of Health, Bethesda, MD, USA; available at http://rsb.info.nih.gov/ij/index.html) (Figures 1 and 2) and calculated the expansion rates from the proportions of the average area of laser scars against the optic disc area. All the measurements were performed twice by one investigator (Masahiko Higaki‘s visual inspection on clopped magnified images). Results were obtained by analyzing the mean values of the two measurements. The intraclass correlation coefficient (ICC, %) was also calculated to evaluate reproducibility.

The regressions of neovascularization were evaluated by fluorescein angiography (FA). FA was performed 6 months and 12 months after treatment to evaluate the efficacy of photocoagulation, and if there were any residual nonperfusion area or neovascularization, additional laser photocoagulation was applied.

### 2.1. Statistics

All results are expressed as the mean ± standard deviation. Differences in genders and severity of diabetic retinopathy were analyzed by the Fisher's exact test. Comparisons of age, BCVA, CMT, timing of additional laser, and the duration of follow-up were performed using the Student's *t*-test. Expansion rates were analyzed using repeated measure ANOVA. The number of PRP shots was compared with Mann–Whitney *U* test. In all analyses, *p* < 0.05 was considered to be statistically significant. Statistics were calculated using Statcel 3 statistical software, version 3 (OMS Inc., Saitama, Japan).

## 3. Results

### 3.1. Patient Characteristics

The laser treatment was performed with the conventional laser (Novus Varia, Lumenis, Santa Clara, CA, USA) in 6 eyes and the short-pulse laser (PASCAL Streamline) in 11 eyes. Clinical characteristics of the patients are shown in Table 1. Conventional laser group included 3 PDR eyes, and short-pulse laser group included 2 PDR eyes. Although the conventional laser group included more PDR eyes, there was no statistically significant difference.

The mean age of patients was 65.8 ± 8.3 (range: 53–77) years old in the conventional laser group and 55.0 ± 14.1 (range: 34–77) years old in short-pulse laser group. The mean follow-up period was 15.5 ± 3.6 (range: 12–21) months in the conventional group and 16.6 ± 3.7 (range: 12–24) in short-pulse laser group. There were no statistically significant differences in age and follow-up period between the two groups. And all phakic patients did not receive cataract surgery during the follow-up period.

### 3.2. Laser Setting Parameters

Both laser methods were performed in the same spot size (200 *μ*m) at different power to attain gray-white burn with Mainster PRP 165 contact lens (Ocular Instruments Inc., Bellevue, WA, USA). Yellow wavelength (577 nm) was used in both modalities. Sub-Tenon's triamcinolone acetonide (Kenacort; Bristol-Myers Squibb, Tokyo, Japan) injections (STTA) were performed after the first session of laser treatment (4 eyes in the conventional laser group and 2 eyes in the short-pulse laser group). The summary of the settings used in the conventional laser and the short-pulse laser was shown in Table 2. One eye in the conventional laser group was previously treated with targeted retinal photocoagulation (TRP) [20]. In the short-pulse laser group, 4 eyes were treated with TRP, and 3 eyes were previously treated with TRP. Other 4 eyes were treated with PRP. The mean PRP number of laser shots performed in the treatment-naive eye was 1798 ± 885 in the conventional laser group and 4247 ± 279 in short-pulse laser group, and there was a significant difference (*p* < 0.05, Mann–Whitney *U* test).

### 3.3. The Best-Corrected Visual Acuity

The mean BCVA (logMAR) before the conventional laser treatment was 0.64 ± 0.41 and 0.35 ± 0.44 at 12 months after treatment. The mean BCVA before the short-pulse laser treatment was −0.05 ± 0.12 and 0.00 ± 0.13 at 12 months after treatment. There was no significant aggravation of BCVA 12 months after treatment in both groups.

### 3.4. Central Macular Thickness (CMT)

The mean CMT before the conventional laser treatment was 339.6 ± 80.0 *μ*m, and the mean CMT at 12 months after treatment was 329.0 ± 81.0 *μ*m. The mean CMT before the short-pulse laser treatment was 266.5 ± 35.4 *μ*m and that at 12 months after treatment was 272.1 ± 32.3 *μ*m. There was no significant aggravation of CMT 12 months after treatment in both groups.

### 3.5. Disease Regression Outcomes

In the conventional laser group, two eyes (33%) required additional laser due to the residual nonperfusion area (9 or 13 months after treatment). Two eyes (33%) were treated additionally due to residual nonperfusion area and neovascularization (7 or 13 months after treatment). And one eye (14%) developed macular edema 4 months after laser treatment, and focal laser photocoagulation was performed using Navilas laser system (OD-OS GmbH, Teltow, Germany). This patient was previously treated by several injections of antivascular endothelial growth factor (VEGF) for diabetic macular edema (DME). At the time when PRP was given, DME was resolved, and she was not treated with STTA.

In the short-pulse laser group, 4 eyes (36%) received additional laser due to the residual nonperfusion area (6–9 months after treatment). One eye developed retinal break with posterior vitreous detachment, and the laser photocoagulation was performed around the retinal break (5 months after treatment). One eye (9%) showed recurrence of macular edema 7 months after laser treatment, and focal laser photocoagulation was performed. This patient was treated with STTA when PRP was given (4411 shots in one session).

The timing of additional laser showed no significant difference between both groups.

### 3.6. Photocoagulation Scar Expansion

We measured the size of 20 laser scars near the vascular arcade on each visit and calculated the expansion rate over time. The intraclass correlation coefficient (ICC, %) was evaluated (Table 3). Based on these results, the collected data were considered to be reliable and useful for further analysis.

**Figure 3:**
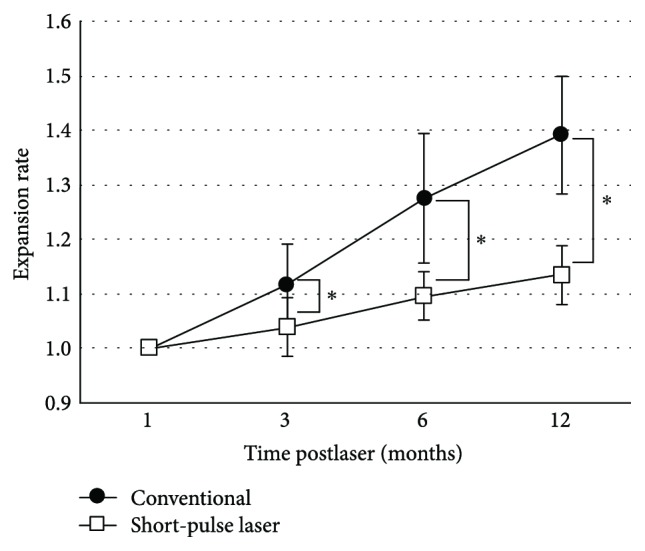
Expansion rates of laser scars with the conventional laser (closed circle) and short-pulse laser (open square) in months 3, 6, and 12 after laser treatment. Laser scars significantly expanded in both modalities, and the expansion rates of the short-pulse laser were significantly lower than those of conventional laser scars. Repeated measure ANOVA,^∗^*p* < 0.01.

**Figure 4:**
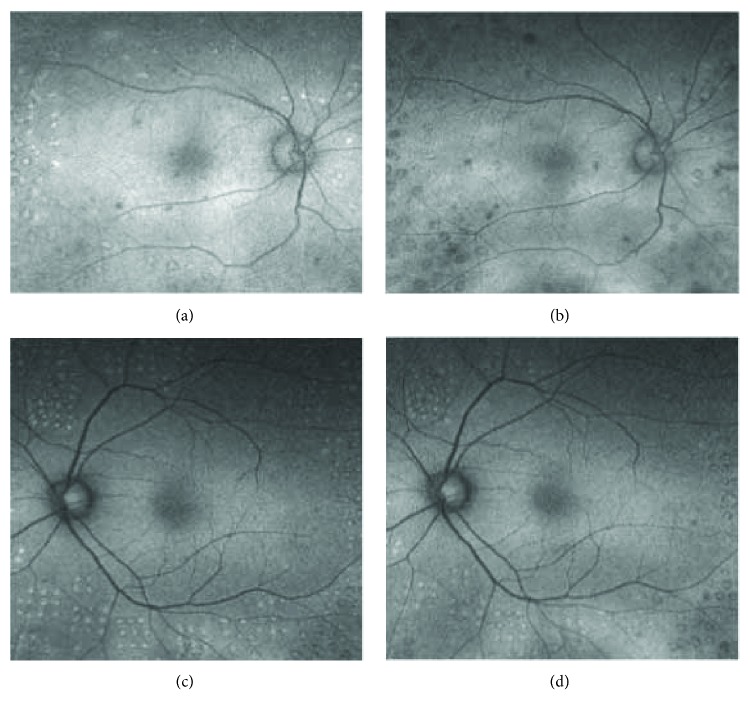
Representative images of FAF in the conventional laser group (a, b) and short-pulse laser group (c, d). The images were taken 3 months after laser treatment (a, c) and 6 months after laser treatment (b, d). Three months after laser treatment, the laser scars showed an increased level of autofluorescence (AF) surrounded by a decreased level of AF in both groups (a, c). Six months after laser treatment, the laser scars in the conventional laser group changed to hypoautofluorescent (b). However, in the short-pulse laser group, laser scars did not change to hypoautofluorescent (d).

The expansion rates of scars with the conventional laser were 1.12 ± 0.08 (3 M), 1.27 ± 0.12 (6 M), and 1.39 ± 0.11 (12 M) (Figures 3 and 4).

On the other hand, the expansion rates of scars with the short-pulse laser against the scar size in 1 month after treatment were 1.04 ± 0.05 (3 M), 1.09 ± 0.04 (6 M), and 1.13 ± 0.05 (12 M) (Figures 3 and 4).

As a result, the expansion rates of both groups increased significantly over time (*p* < 0.01) and the expansion rates of short-pulse laser scars were significantly lower than those of conventional laser scars over time (*p* < 0.01) (Figure 3). There were 5 operators in each group, and there were no significant differences in expansion rates among operators.

### 3.7. FAF Findings

The conventional laser scars changed from hyperautofluorescent to hypoautofluorescent more rapidly than the short-pulse laser scars (Figure 4). All the photocoagulation scars in both groups were hyperautofluorescent at month 3 (Figures 4(a)–4(c)). The photocoagulation scars in the conventional laser group became hypoautofluorescent in 5 out of 6 eyes (83.3%) at month 6 (Figure 4(b)) and 6 out of 6 eyes (100%) at month 12. On the other hand, the photocoagulation scars in the short-pulse laser group became hypoautofluorescent in 1 out of 11 eyes (9.1%) at month 6 (Figure 4(d)) and 7 out of 11 eyes (63.6%) at month 12. There was no relationship between FAF changes and laser augmentations. The FAF findings were all similar among operators.

## 4. Discussion

Our study showed that the laser photocoagulation scars kept growing for 12 months; however, the expansion rates of the short-pulse laser scars were significantly lower than those of the conventional laser scars during the period of observation. We used noninvasive FAF images taken by Optos 200Tx. By using FAF images, we were able to measure the sizes of photocoagulation scars easily due to their sharp outlines [15, 16].

These results were consistent with the following two previous reports. According to Nagpal et al., the expansion rate of the conventional laser was 27.2% and that of the short-pulse laser was 14.0% three months after laser treatment [8]. Shiraya et al. showed us that the expansion rate of the conventional laser was 18% and that of the short-pulse laser was 14% six months after laser photocoagulation [12].

On the other hand, some reports indicated that the 20-millisecond-pulse burns progressively reduced in size after photocoagulation [9, 15, 16, 21]. It can be surmised that the early retinal edema decreased with the lapse of time in these reports. Therefore, we set the values in one month after laser treatment as a benchmark to avoid the effect of the early retinal edema.

When the conventional laser is performed, photoreceptors usually suffer damage although the main target is RPE. Photoreceptors connect with adjacent photoreceptors through horizontal or amacrine cells. After local photoreceptors undergo necrosis, it causes apoptosis of the surrounding photoreceptors subsequently. As a consequence, photocoagulation scars expand [11, 22]. In contrast, when the short-pulse laser is performed, the retinal damage is mostly confined to the outer retina because its pulse duration is very short (10–30 ms) [16, 21, 22]. Accordingly, photoreceptors suffer much less damage and the photocoagulation scars enlarge less than the conventional laser as a result.

In this FAF study, the short-pulse laser scars changed from hyperautofluorescent to hypoautofluorescent more slowly than the conventional laser scars. In the short-pulse laser group, all 4 eyes followed by 18 months showed hypoautofluorescent scars. This is possibly because of the chorioretinal damage by the short-pulse laser is confined to the outer retina. Conventional laser induces choriocapillaris atrophy which accelerates death of RPE and photoreceptors [23] and accelerated death of RPE and photoreceptors resulted in reduced FAF signal [19].

These results should be considered when laser photocoagulation therapy is performed for patients who have diabetic retinopathy or other retinal diseases. A report indicated that PRP performed by the short-pulse laser is less effective than that performed by the conventional argon laser in regression of neovascularization or incidence of vitreous hemorrhage within 6 months after treatment when the same number of spots was applied [5]. It is possible to deduce that the total area of PRP scars in the argon-treated patient exceeds that of the patient who underwent the short-pulse laser [10], and we should also consider the variability of photocoagulation lesions between physicians and patients [24], although there were no differences in expansion rates among operators in this study. Therefore, it is important for an operator to reconsider the settings of treatment parameters when using short-pulse laser therapy for serious retinal diseases such as high-risk PDR [5]. In this study, we set spacing as 0.75, and the total number of laser spots for PRP is significantly higher in the short-pulse laser group, and during this study follow-up period, no eye developed new vitreous hemorrhage in both groups during this study. Although the number of eyes with PDR was higher in the conventional laser group, suitable space setting (0.75) and higher number of laser spots might result in successful PRP.

As for PRP-induced macular edema, there were no significant differences in CMT before and after laser photocoagulation both in the conventional laser and short-pulse laser group in this study. STTA before PRP has been known as an effective treatment to prevent from PRP-induced macular edema [25], and we usually employ STTA when we start PRP in the eyes with already existing macular edema. However, one eye developed macular edema after PRP in the conventional laser group. She had past history of DME treated with multiple injections of anti-VEGF, but she did not receive STTA when PRP was given because macular edema was resolved at that time. But her parafoveal retinal thickness was 372 *μ*m when PRP was initiated. Shimura et al. reported that patients whose preoperative parafoveal thickness was >300 *μ*m had a worse visual prognosis due to PRP-induced macular edema [26]. From this background, this eye also should have been treated with STTA when PRP was given. Conversely, one eye also showed recurrence of macular edema after PRP in the short-pulse group 7 months after treatment, and he was treated with STTA when PRP was given (4411 shots). From the overall results, there was no difference in terms of regression of retinopathy and worsening of macular edema between the conventional laser group and short-pulse laser group in our study.

There were several limitations that need to be acknowledged in our current study. First, there was a relatively small number of eyes with nonrandomized, retrospective methods. To compare the efficacy and expansion rate with the short-pulse laser and conventional laser, a large number of study with randomization will be warranted. Second, we used the wide-field imaging system, but we adopted only the postpole area. The reason was because the magnification of the posterior pole and that of midperiphery was different when using the images of Optos 200Tx [27]. Moreover, laser photocoagulation scars enlarge more in the posterior pole area than in the peripheral area [11]. Taking these differences into consideration, we decided to adopt the photocoagulation scars to evaluate only in the area of the posterior pole. Recently, the new software using stereographic projection, in which the lesion areas on ultra-wide-field images can be calculated in anatomically correct physical units (mm^2^), has been developed [28]. Nevertheless, this software is not commercially available yet, we believe that the total area of laser scar evaluation using FAF will give us more useful information of efficacy on laser photocoagulation in the future.

## 5. Conclusion

FAF imaging was useful to evaluate the temporal changes in the laser photocoagulation scar size. The scars with the short-pulse laser consistently showed lower expansion rates compared with those of the conventional laser. The change in CMT between the two groups was not significant.

## Figures and Tables

**Table 1:** Patient characteristics.

	Conventional laser	Short-pulse laser	*p*
Number of eyes	6	11	
Mean age, years	65.8 ± 8.3	55.0 ± 14.1	0.13†
Male : Female	4 : 2	5 : 3	0.65‡
NPDR : PDR	3 : 3	9 : 2	0.29‡
Duration of follow-up (months)	15.5 ± 3.6	16.6 ± 3.7	0.58†
BCVA (logMAR) pretreatment	0.64 ± 0.41	−0.05 ± 0.12	<0.01†
BCVA (logMAR) posttreatment (12 M)	0.35 ± 0.44	0.00 ± 0.13	<0.05†
Mean CMT pretreatment (*μ*m)	339.6 ± 80.0	266.5 ± 35.4	<0.05†
Mean CMT post- treatment (*μ*m) (12 M)	329.0 ± 81.0	272.1 ± 32.3	0.08†
Phakic eyes : pseudophakic eyes	3 : 3	10 : 1	0.10‡
Number of operators	5	5	
STTA	4	2	0.07‡

†Student's *t*-test; ‡Fisher's exact test. NPDR: nonproliferative diabetic retinopathy; PDR: proliferative diabetic retinopathy; BCVA: best-corrected visual acuity; CMT: central macular thickness; STTA: sub-Tenon's injections of triamcinolone acetonide.

**Table 2:** Settings of laser treatment.

	Conventional laser	Short-pulse laser	*p*
Power (mW)	100–260	300–500	—
Pulse duration (ms)	200	20	—
Spot size (*μ*m)	200	20	—
Wavelength (nm)	Yellow (577)	Yellow (577)	—
Spacing (spot)	1	0.75	—
Mean number of total PRP shots (in treatment naive eyes)	1798 ± 885 (958–3505)	4247 ± 279 (3875–4600)	<0.05†

†Mann–Whitney *U* test. PRP: panretinal photocoagulation.

**Table 3:** Intraclass correlation coefficient.

	Conventional group		Short-pulse group	
	M1	M2	M1	M2
Mean scar area divided by disc area, month 1	0.072 ± 0.010	0.074 ± 0.009	0.037 ± 0.040	0.038 ± 0.003
Mean of M1 and M2	0.073 ± 0.009		0.037 ± 0.004	
ICC, %	81.6		79.2	
Mean scar area divided by disc area, month 3	0.081 ± 0.013	0.081 ± 0.012	0.039 ± 0.004	0.040 ± 0.004
Mean of M1 and M2	0.081 ± 0.013		0.040 ± 0.004	
ICC, %	88.3		81.1	
Mean scar area divided by disc area, month 6	0.093 ± 0.012	0.093 ± 0.011	0.041 ± 0.004	0.041 ± 0.004
Mean of M1 and M2	0.093 ± 0.011		0.041 ± 0.004	
ICC, %	87.0		87.3	
Mean scar area divided by disc area, month 12	0.102 ± 0.017	0.102 ± 0.016	0.043 ± 0.003	0.043 ± 0.003
Mean of M1 and M2	0.102 ± 0.016		0.043 ± 0.003	
ICC, %	83.5		84.1	

M1: measurement 1; M2: measurement 2. ICC: intraclass correlation coefficient.

## Data Availability

The data used to support the findings of this study are available from the corresponding author upon request.
